# Independent early predictors of mortality in polytrauma patients: a prospective, observational, longitudinal study

**DOI:** 10.6061/clinics/2017(08)02

**Published:** 2017-08

**Authors:** Luiz Guilherme V. da Costa, Maria José C. Carmona, Luiz M. Malbouisson, Sandro Rizoli, Joel Avancini Rocha-Filho, Ricardo Galesso Cardoso, José Otávio C. Auler-Junior

**Affiliations:** I Divisao de Anestesiologia, Hospital das Clinicas HCFMUSP, Faculdade de Medicina, Universidade de Sao Paulo, Sao Paulo, SP, BR; II Grupo de Resgate e Atendimento as Urgencias (GRAU), Secretaria de Estado da Saude, Sao Paulo, SP, BR; III University of Toronto Trauma and Acute Care Service, St Michael’s Hospital, Toronto, Canada

**Keywords:** Multiple Trauma, Indicators, Mortality, Shock, Brain Injuries, Anoxia

## Abstract

**OBJECTIVES:**

Trauma is an important public health issue and associated with substantial socioeconomic impacts and major adverse clinical outcomes. No single study has previously investigated the predictors of mortality across all stages of care (pre-hospital, emergency room, surgical center and intensive care unit) in a general trauma population. This study was designed to identify early predictors of mortality in severely injured polytrauma patients across all stages of care to provide a better understanding of the physiologic changes and mechanisms by which to improve care in this population.

**METHODS:**

A longitudinal, prospective, observational study was conducted between 2010 and 2013 in São Paulo, Brazil. Patients submitted to high-energy trauma were included. Exclusion criteria were as follows: injury severity score <16, <18 years old or insufficient data. Clinical and laboratory data were collected at four time points: pre-hospital, emergency room, and 3 and 24 hours after hospital admission. The primary outcome assessed was mortality within 30 days. Data were analyzed using tests of association as appropriate, nonparametric analysis of variance and generalized estimating equation analysis (*p*<0.05). ClinicalTrials.gov: NCT01669577.

**RESULTS:**

Two hundred patients were included. Independent early predictors of mortality were as follows: arterial hemoglobin oxygen saturation (*p*<0.001), diastolic blood pressure (*p*<0.001), lactate level (*p*<0.001), Glasgow Coma Scale score (*p*<0.001), infused crystalloid volume (*p*<0.015) and presence of traumatic brain injury (*p*<0.001).

**CONCLUSION:**

Our results suggest that arterial hemoglobin oxygen saturation, diastolic blood pressure, lactate level, Glasgow Coma Scale, infused crystalloid volume and presence of traumatic brain injury are independent early mortality predictors.

## INTRODUCTION

Trauma remains the leading cause of mortality and severe disability in adults and constitutes a major public health problem and important subject of scientific research in different areas of clinical practice, such as prevention, intensive care and rehabilitation 1-3. External causes have been estimated to account for numerous hospitalizations in the public health system in Brazil, mainly comprising road traffic accidents and homicides. When analyzing data from 2007, it was estimated that violence and injuries accounted for 12.5% of all deaths in Brazil 4. Despite the extensive human and socio-economic impact associated with traumatic injuries, no previous studies have prospectively identified independent predictors of mortality across the entire spectrum of trauma care (pre-hospital, emergency room, operating room and intensive care unit phases) within the same trial 5. Most studies have focused on isolated stages in the spectrum of care 6, 7 or on specific types of trauma (e.g., traumatic brain injury (TBI), pelvic trauma, and penetrating torso injuries) 8-12. Other reports have analyzed large databases and prognostic models 8, 13, 14; however, no studies have assessed the determinants of mortality across all the stages or phases of care at the same time.

The metropolitan city of São Paulo has an area of 8.5 km^2^, a population of more than 20 million inhabitants and an average of 11,379 cases per month attended to by pre-hospital rescue system teams. The provision of care to and rehabilitation of this large contingent of polytrauma patients has been associated with a large socioeconomic burden 5. In this context, numerous questions exist regarding the physiologic changes that occur from the earliest moments of trauma (pre-hospital) to the later stages of trauma (intensive care unit). These changes serve as the major factors that should be investigated to understand the standard of care provided to and improve outcomes in this population. The findings (clinical and epidemiological findings) related to mortality predictors that have been presented in the trauma literature thus far have been highly heterogeneous, and a variety of study designs have been employed to assess these data with conflicting results 9,11,14-18 observed between centers 12.

Considering this scenario, a longitudinal, prospective, observational study was conducted between 2010 and 2013 to examine victims of severe trauma in the aforementioned region. The study was designed to assess the overall care provided to these patients from the pre-hospital phase to the final phase of hospital care.

The objective of this study was to identify independent predictors of mortality in severe trauma patients across the entire spectrum of care, from the earliest stage of care in the pre-hospital setting to admission to the intensive care unit (ICU) and hospital discharge. Additionally, the demographic profile of the studied population was determined.

## MATERIALS AND METHODS

The study protocol was approved by the Institutional Medical Ethics Committee (CAPPesq 1081/09) and was conducted in accord with the Helsinki Declaration of 1975 (revised in 1983). Financial support was received from the São Paulo State Research Foundation (Fundação de Amparo à Pesquisa do Estado de São Paulo - FAPESP) under grant no. 2010/03315-4. The protocol is registered on ClinicalTrials.gov (NCT01669577).

The screening strategy included the identification of general trauma patients (>18 years old) submitted to high-energy trauma (potential or identified severe bleeding, severe traumatic brain injury (TBI) [Glasgow coma score (GCS <9)], significant damage resulting from high-velocity car crashes, falls >5 m, gunshots, penetrating torso/abdominal injuries, pedestrian car accidents and traumatic limb amputations) attended to and screened by rescue system medical teams and taken to HCFMUSP (Hospital das Clínicas - University of São Paulo, School of Medicine, Teaching Hospital) by land or helicopter. During data analysis, patients with an injury severity score (ISS) <16 (calculated at the hospital after the abbreviated injury score [AIS] was determined by surgical staff) were excluded.

Exclusion criteria also included lack of written informed consent (provided by the patients after clinical stabilization at hospital, if possible, or from a relative or representative), situations in which data collection could compromise victim care, technical problems during data collection, and insufficient blood samples or data. Patients who died before hospital arrival were not included.

Patients were brought to the hospital by the Air Patrol Division or ambulances of fireman headquarters in São Paulo. This strategy was adopted to identify the majority of severely injured victims in this region and allow for an acceptable time period from scene to hospital (<30 minutes).

Missing data were treated as missing at random (MAR), and appropriate treatment of data was provided 19.

For all patients, data were recorded at the following time points: 1, at the trauma scene; 2, in the emergency room; 3, at 3 hours after hospital admission; and 4, at 24 hours after hospitalization.

Data for gender, age, trauma mechanism and medical procedures performed during each stage, time until arrival at the hospital and comorbidities were recorded. The following clinical data were collected: systolic blood pressure (SBP), diastolic blood pressure (DBP), heart rate (HR), respiratory rate (RR), arterial hemoglobin oxygen saturation (SAT) (measured before supplementary oxygen), Glasgow coma score (GCS), and pupil pattern. All patients received 100% inspired oxygen through a non-rebreathing face mask or tracheal intubation.

The following laboratory data were collected through peripheral venipuncture during each hospital stage: pH level; base excess (BE) value; partial oxygen pressure (pO_2_); partial carbon dioxide pressure (pCO_2_); arterial hemoglobin oxygen saturation; bicarbonate (BIC), lactate, sodium (Na^+^), potassium (K^+^), ionized calcium (Ca^2+^), glucose, hemoglobin (Hb), hematocrit (Ht), and serum creatine phosphokinase (CPK) levels; and platelet and leukocyte counts. Data for volume expansion [crystalloids (CRYSTAL) and colloids (COLO) infused], diuresis and water balance were also collected. The use of blood products [packed red blood cells (PRBC), fresh frozen plasma (FFP), platelet concentrates (PLAT), and cryoprecipitate concentrates (CRYO)] and the use of vasoactive drugs (VAD) were recorded at the corresponding time points. Patient follow-up was conducted in the ICU, and the duration of ICU stay (days in the ICU), time under mechanic ventilation (days under MV), and presence or absence of sepsis (systemic inflammatory response syndrome and confirmed biological agent), coagulopathy^20^ (INR >1.4 or R >1.2) and acute renal failure 21 (ARF) were recorded.

Blood coagulation data were collected as follows: time point 1, international normalized ratio (INR) and prothrombin time (PT); time points 2, 3 and 4, INR, PT, activated partial thromboplastin time (aPTT), the aPTT ratio between patients and controls (R) and thrombin time (TT).

The following severity indexes were calculated: ISS; revised trauma score (RTS); trauma and injury severity score (TRISS); and simplified acute physiology score 3 (SAPS 3) (the latter was determined when the patient was in the ICU).

At time point 1, blood tests were performed using an i-STAT^®^ device (Abbott, USA; PT/INR and CG4^+^ and CG8^+^ kits). During each of the hospital stages, the clinical analysis methodology of the HCFMUSP was used.

Data were collected during the first 24 hours of treatment, and patients were clinically followed up for 30 days.

All patients received care from senior surgeons, intensivists, anesthesiologists, radiologists and clinicians who were members of the HCFMUSP staff. Tranexamic acid was administered to polytrauma victims when indicated 22. Blood component transfusions were guided by rotational thromboelastometry during each stage of care in our hospital.

### Statistical Analysis

A sample power analysis was conducted with a significance level of 0.05, power of 0.80, moderate correlation of 0.5 between time periods and assumption that variability was equal within each factor (non-sphericity). Due to the calculation of effect sizes between 0.1 and 0.5, there was no need for a sample size larger than 140 patients. Thus, a total of 200 patients was conservatively defined, with a margin of error included to account for the possibility of death. G*Power 3.1.7 software was used for sample size calculation.

Data analysis was divided into three interconnecting parts. The first part utilized descriptive data analysis and tests of association between independent variables and death. The second part addressed the profiles of time-dependent measures and their relationships with death through analyses of nonparametric variance for repeated measures. The third part evaluated the results of all previous analyses, and a generalized estimating equation (GEE) was constructed.

For the first part of the analysis, both the overall group (n=200) and two subgroups, namely, patients who died (n=52) and those who survived (n=148), were assessed. For categorical variables, the two-tailed Fisher’s exact test was used, and for continuous variables, the two-tailed *t* test or the two-tailed Mann-Whitney test was used according to the normality of the variable, which was verified using the Anderson-Darling test.

In the second part of the analysis, the longitudinal profiles of each measure were analyzed for the subgroups of survivors and non-survivors. Nonparametric analyses of variance (NPar ANOVA) were conducted.

Finally, in the third part of the analysis, a GEE model was developed considering the family of binomial distributions (dichotomous response variable) with the logit link function. Only the main effects of each measure were considered. The NPar ANOVA test (*p*<0.1 for death) was used to identify variables for inclusion in the model. The variables included in the final models were selected using the backward selection method with an output alpha equal to 0.05. All results from part three were interpreted by estimating odds ratios (ORs), corresponding 95% confidence intervals and significance tests (*p*-value).

The significance level was set at 0.05, and the free R 3.0.2 software was used to perform all statistical analyses.

## RESULTS

**Figure 1:**
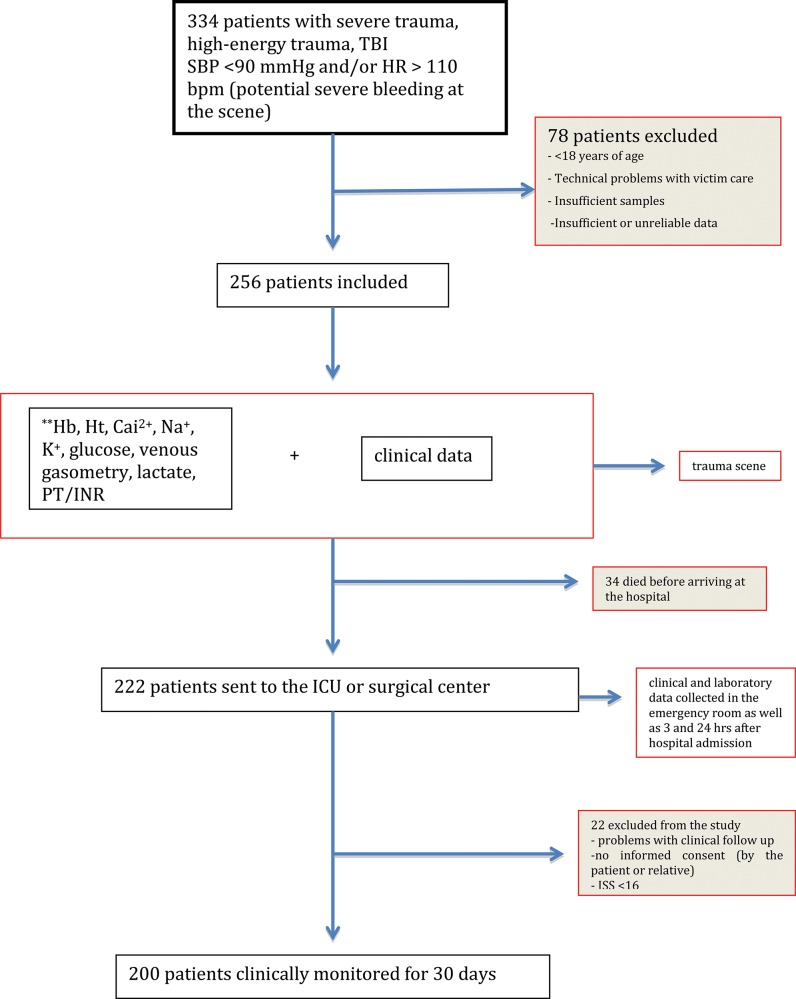
Flowchart. ISS - injury severity score; SBP - systolic blood pressure; Hb - hemoglobin (g/dL); Ht - hematocrit (%); PT/INR - prothrombin time (s)/international normalized ratio; Ca^2+^ - ionized calcium (mmol/L); Na^+^ - sodium (meq/L); K^+^ - potassium (meq/L).

Using the previously described screening strategy, 334 trauma patients were identified. In total, 78 patients were excluded because they did not meet the inclusion criteria, leaving 256 patients for inclusion in the analysis. Of these patients, 34 patients died before arriving at the emergency room, and 22 patients were removed from the analysis due to incomplete data (Figure 1).

Among the 200 included patients, a mortality rate of 26% (n=52) was observed, and survivors were followed up for 30 days.

The descriptive data showed a predominance of males (n=164; 82%), a mean age of 37.3 years (standard deviation=14.63) and a high prevalence of TBI (n=130; 65%) among patients (Tables 1 and 2).

**Figure 2:**
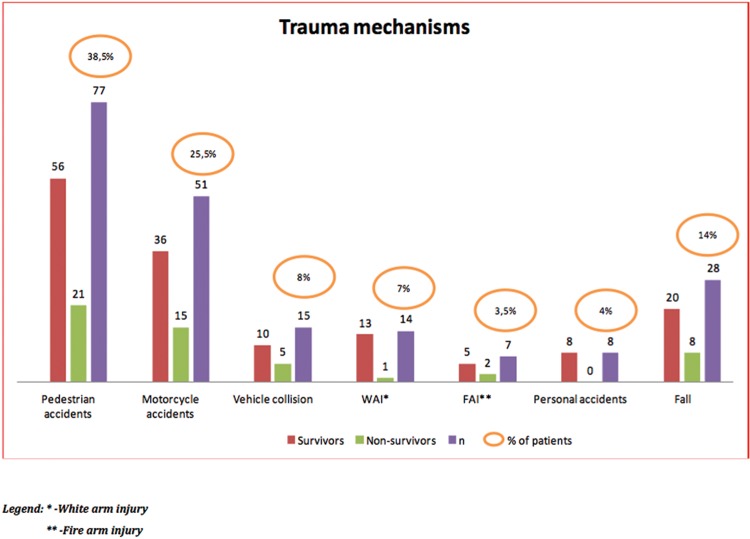
Comparison of the absolute numbers of the types of trauma mechanisms between the survivor and non-survivor groups.

Regarding the trauma mechanisms, there was a predominance of pedestrian accidents (38.5%), followed by accidents involving motorcycles (25.5%) and falls (14%) (Figure 2).

The variables that differed significantly over time relative to their associated odds of death (included in GEE analysis) were as follows: SAT (*p*<0.001), SBP (*p*<0.017), DBP (*p*<0.001), pH level (*p*=0.002), BE value (*p*=0.002), bicarbonate level (*p*=0.005), lactate level (*p*<0.001), partial CO_2_ pressure (*p*=0.025), partial O_2_ pressure (*p*=0.013), GCS score (*p*<0.001), diuresis (*p*=0.008), PT (*p*<0.001), INR (*p*<0.001), aPTT (*p*<0.001), TT (*p*<0.001), and CRYSTAL (*p*<0.027). Due to the vast quantity of data considered at this time point, we listed the variables included in the GEE model based on their main effects and significant association with death in Table 3 (see next topic).

One patient had thrombophlebitis of the forearm, and intravenous access was established. Two patients presented local infections at the peripheral venipuncture site.

### GENERALIZED ESTIMATING EQUATIONS

Gender, age, ISS (16–24, moderate; 25–75, critical) and presence of TBI were initially fixed as control variables in the GEE model.

Table 4 refers to the GEE models, which considered the main effects of the variables. The following variables were significantly associated with death: SAT, DBP, lactate level, GCS, CRYSTAL and TBI.

Data analysis showed that a 1% increase in SAT was associated with a 1.2% decrease in the odds of death, and an increase of 1 mmHg in DBP was associated with a 0.3% decrease in the odds of death. For lactate level, an increase of 1 mmol/L was associated with a 6% increase in the odds of death, while a one-point increase in GCS score was associated with a 2% decrease in the odds of death, and an increase of 1000 mL in CRYSTAL was associated with a 1.6% increase in the odds of death. TBI presence increased the death probability by 508.7% [as expected from previous reports 1, 8, 12].

## DISCUSSION

When considering the characteristics of the present sample, which included data derived based on repeated variable measurements performed at different points defined over time, a more robust statistical method was selected to eliminate errors that may have accumulated in the analytic process and identify potential predictors that would not be determined with the desired accuracy using simpler models. Thus, the use of a GEE model was justified. Using this model, predictive factors were identified that, when combined over time, were significantly associated with patient mortality 23. This method allows an adequate adjustment of covariates between groups of survivors and non-survivors, as it takes into account correlations between repeated measures. In addition, this methodology does not require data to be distributed in any particular manner and allows for the computation of robust estimates.

The findings of the GEE analysis (Table 4) showed that the independent predictors of mortality were SAT (OR=0.988), DBP (OR=0.997), lactate level (OR=1.06), GCS score (OR=0.980), CRYSTAL (OR=1.016/1000 ml infused) and presence of TBI (OR=6.087).

SAT, as previously described, plays a crucial role in maintaining brain function and may have been especially important given the high number of TBIs affecting the patients in this study. In the present sample, a 1% increase in SAT was associated with a 1.2% decrease in mortality, and hypoxemia was a determining factor for poor clinical outcomes in this population 12, 24-26.

DBP plays a vital role in the perfusion of key organs, such as the heart and brain. The present study found that an increase of 1 mmHg in DBP was associated with a 0.3% decrease in the probability of death. Unlike other studies that have cited SBP as a hemodynamic indicator 9,27-30 and predictor of mortality, in the present study, it was demonstrated that DBP (and not SBP) ultimately appeared to be a more representative value for clinical prognosis 31, 32 and thus deserves further investigation. Perhaps this finding is due to the pivotal role of DBP in mean arterial pressure, which has been widely studied as a factor associated with the coronary and cerebral perfusion pressure gradient 25,30,32,33. In addition, left heart perfusion depends greatly on diastolic pressure 27.

In this study, lactate range had an important impact on mortality in polytrauma patients, and similar results have been observed in several other clinical settings 34-39; thus, patient lactate level should be monitored early on, as it can serve as an indicator of microcirculatory perfusion and progression to multiple organ failure 35,36, 40,41. In the present study, an increase of 1 mmol/L in lactate level was associated with a 6% increase in the probability of death.

A patient’s GCS reflects the severity of TBI and may be representative of the probability of death after polytrauma 13, 17,42,43. Because of its extreme significance, GCS should be used as a warning sign to guide the performance of a rigorous investigation for and facilitate the resolution and prevention of secondary injuries 44. The association between mortality and GCS score in the present study suggested that each increased GCS point corresponded to a 2% decrease in mortality, in accordance with prior literature on the subject 43.

CRYSTAL appeared to be a silent mortality indicator during resuscitation following severe trauma. The infusion of a large amount of crystalloids during the first 24 hours is common and, because of the hemodynamic instability of trauma victims, caution is required regarding the volume of fluids infused 15, 45 to avoid a large accumulated water volume, which is a known indicator of morbidity and mortality 46,47. In the present study, each 1000 mL of crystalloid infused was associated with an increase of 1.6% in the probability of mortality. This finding contradicts recent findings regarding fluids and trauma resuscitation 16 and reaffirms classic findings suggesting that a lower volume of fluid reposition is associated with a decrease in the rate of mortality in this population 11,15, 45-47. Whereas other studies have aimed to show that the administration of fluids, regardless of type, may be a surrogate indicator for sickness and mortality 48, our analysis suggested a clear relationship between crystalloid volume infused and mortality, as the GEE model was adjusted for both ISS and TBI presence. This finding is very important, as an infusion of 15 to 20 liters of crystalloids after 24 hours of trauma care in severely injured patients may be easily achieved (in this case, the aggregated mortality would be 24% to 32%).

The presence of TBI greatly increased the probability of mortality (508.7%), in accordance with previous reports 12. In addition, this result could unmask potential confounders such as GCS once patients are sedated, reducing its value over time.

Interestingly, our results did not indicate that mortality risk was associated with the transfusion of blood components. This finding could be explained by the institutional practice of thromboelastometry-guided transfusion and tranexamic acid administration, which can improve the care provided to patients with massive bleeding and consequently balance risk between survivors and non-survivors 22.

The administration of colloids in the present study was minimal (10 patients), and its influence on mortality was not significant.

This study was conducted in a region with an ethnically diverse patient population affected by multiple trauma mechanisms (mostly high-energy trauma), a male predominance and a high incidence of TBI (65%), a characteristic previously observed in other studies 8, 9, 22, 28, 43, 49. Therefore, there may have been an implicit bias in the findings and a limitation due to the sampling characteristics described. However, these characteristics are common to large trauma centers around the world, which tend to receive extremely injured trauma patients. Another limitation of this study lies in the fact that its cohort was based on a single trauma center.

A unique characteristic of our study was the integrated evaluation of predictors of mortality across all stages of trauma care, from the pre-hospital phase (which has been neglected in the majority of trauma literature) to the emergency room, surgical center, ICU and thirty days after hospital discharge. In addition, our study included not only TBI patients 8, 43 but also those who experienced massive bleeding 22 (blunt and penetrating injuries) and severe polytrauma. Data were collected prospectively and analyzed using a sophisticated method, focusing on all stages of care in an integrated fashion rather than on one specific stage. This information will certainly contribute to future meta-analyses and systematic reviews and could help in the generation of prognostic models 13, 43, 50 to fully understand the physiopathology and main targets of trauma care. Further prospective controlled multicentric studies focusing on the factors identified as independent predictors of mortality should be conducted using similar methodology in other world metropolises to generalize and validate the findings of the present study.

After an extensive process of data screening, collection, recording and analysis, arterial oxygen saturation, diastolic blood pressure, serum lactate, Glasgow Coma Scale score, total amount of crystalloid solution infused and presence of traumatic brain injury were found to be independent early predictors of mortality in severe trauma patients. Considering trauma as an important epidemiological factor for mortality in urban centers with a substantial socioeconomic impact in all nations of the world, the findings from this novel integrated and global data analysis will certainly help clarify confusing results and bias in trauma care. Future prognostic models should include these findings in order to create strategies aimed at reducing morbidity and mortality in this population.

## AUTHOR CONTRIBUTIONS

Auler-Junior JO, Carmona MJ, Malbouisson LM, Rizoli S, Rocha-Filho JA and Costa LG contributed to the study concept and design. Costa LG and Malbouisson LM contributed to data analysis and processing. Carmona MJ, Auler-Junior JO, Malbouisson LM and Rizoli S contributed to manuscript writing. Costa LG and Cardoso RG actively participated in data collection and prospecting. Auler-Junior JO, Carmona MJ, Malbouisson LM, Rizoli S, Rocha-Filho JA, Cardoso RG. and Costa LG contributed to the critical revision of the manuscript.

## Figures and Tables

**Table 1:** Comparison of quantitative variables between the survivor and non-survivor groups.

Variable	Survivors (S)	Non-survivors (NS)	p*	n (S/NS)
	Mean (SD)	Mean (SD)		
Age	36.96 (13.86)	38.21 (16.76)	0.843	(148/52)
ISS	29.93 (10.52)	43.88 (17.77)	<0.001	(148/52)
RTS	6.19 (1.52)	4.46 (1.79)	<0.001	(148/52)
TRISS	73.66 (28.25)	36.47 (34)	<0.001	(148/52)
SAPS3	43.68 (12.81)	54.42 (16.83)	0.004	(131/26)
PRBC	2.53 (3.54)	3.38 (4.33)	0.999	(148/50)
FFP	1.45 (2.89)	2.26 (3.87)	0.213	(148/50)
PLAT	0.92 (2.7)	1.84 (3.69)	0.066	(148/50)
Cryoprecipitate	0.18 (1.28)	0.6 (3)	0.603	(147/50)
MV days	5.62 (5.87)	8.96 (14.17)	0.088	(148/52)
ICU days	11.48 (12.41)	9.29 (16.13)	<0.001	(148/52)

SD – standard deviation; ISS – injury severity score; RTS – revised trauma score; TRISS – trauma and injury severity score; SAPS3 – simplified acute physiologic score 3; PRBC – packed red blood cells; FFP – fresh frozen plasma; PLAT – platelet concentrate; MV days – days under mechanical ventilation; ICU days – days in intensive care unit.

* significance level *p*<0.05.

**Table 2:** Comparison of the absolute numbers of qualitative variables between the survivor and non-survivor groups.

Variable	Survivors	Non-survivors	p*
Gender (male/female)	(125/23)	(39/13)	0.144
Sepsis	60	18	0.51
ARF	28	17	0.054
Coagulopathy	56	36	<0.001
TBI	84	46	<0.001
VAD at time point 2	12	19	<0.001
VAD at time point 3	60	26	0.002
VAD at time point 4	60	20	0.007

ARF – acute renal failure; TBI – traumatic brain injury; VAD – vasoactive drug use.

* significance level *p*<0.05.

**Table 3:** Comparison of quantitative variables (significant for death in GEE) between the survivor and non-survivor groups at the time points of standard collection (significant for death at GEE model).

Variable	Time point	Survivors (S) Mean (SD)	Non-survivors (NS) Mean (SD)	p*	n (S/NS)
**SAT (%)**	1	92.62 (7.57)	84.15 (13.88)	<0.001	(148/52)
	2	95.28 (4.62)	88.53 (11.51)		(148/47)
	3	96.37 (3.83)	93.44 (9.34)		(148/32)
	4	97.07 (2.55)	95.56 (4.86)		(148/25)
**DBP (mmHg)**	1	58.05 (24.03)	44.44 (23.56)	<0.001	(148/52)
	2	67.79 (20.68)	48.53 (25.44)		(148/51)
	3	64.14 (13.4)	56.33 (20.29)		(148/33)
	4	70.42 (14.36)	65.6 (13.78)		(148/25)
**Lactate (mmol/l)**	1	4.47 (2.44)	6.83 (3.82)	<0.001	(148/52)
	2	4.42 (3.25)	7.04 (3.92)		(148/50)
	3	3.97 (3.1)	7.01 (5.25)		(148/33)
	4	3.5 (4.34)	5.29 (4.42)		(148/25)
**CRYSTAL (ml)**	1	823.99 (793.86)	1016.67 (707.79)	0.027	(148/51)
	2	1382.21 (1217.49)	1266.67 (1208.58)		(148/51)
	3	3121.68 (2608.27)	4627.44 (4034.33)		(148/34)
	4	1815.74 (2127)	2535.15 (2186.96)		(148/27)
**GCS**	1	10.44 (4.5)	6.13 (4.03)	<0.001	(148/52)
	2	9.22 (4.92)	4.98 (3.67)		(148/51)
	3	5.16 (3.91)	3.82 (2.48)		(148/33)
	4	7.38 (4.46)	4.16 (2.15)		(148/25)

SD – standard deviation; SAT – arterial hemoglobin oxygen saturation; DBP – diastolic blood pressure; CRYSTAL – infused crystalloid volume; GCS – Glasgow coma score.

* significance level *p* <0.05.

**Table 4:** Generalized estimating equation model for death as a function of the main effects of the measures performed over the 4 time points.

	OR	Lower CI*	Upper CI*	*p* value**
SAT	0.988	0.981	0.995	*p*<0.001
DBP	0.997	0.995	0.999	*p*<0.001
LACTATE	1.060	1.029	1.093	*p*<0.001
GCS	0.980	0.970	0.991	*p*<0.001
CRYSTAL	1.000016	1.000002	1.000031	0.015
TBI	6.087	2.449	15.126	*p*<0.001

OR – odds ratio; CI – confidence interval; SAT – arterial hemoglobin oxygen saturation; DBP – diastolic blood pressure; GCS – Glasgow coma score; CRYSTAL – infused crystalloids volume; TBI – traumatic brain injury.

* 95% confidence interval;

** significance level *p*<0.05.
